# Lycopene Ameliorates Metabolic Dysfunction-Associated Steatotic Liver Disease via PINK1/Parkin-Mediated Mitophagy Activation and Apoptosis Attenuation

**DOI:** 10.3390/antiox15050648

**Published:** 2026-05-21

**Authors:** Ze Xu, Xiao Wu, Lin Ye, Zeqi Li, Jian Zhao, Zhaofeng Zhang, Yongye Sun

**Affiliations:** 1 Department of Nutrition and Food Hygiene, School of Public Health, Qingdao University, Qingdao 266071, China; ze1995@163.com (Z.X.); wxiaoyykx@163.com (X.W.); lizeqi_lizeqi@163.com (Z.L.); zhaojian1@qdu.edu.cn (J.Z.); 2 School of Basic Medicine, Qingdao University, Qingdao 266071, China; a9825711@outlook.com; 3 Department of Nutrition and Food Hygiene, School of Public Health, Peking University Health Science Center, Beijing 100191, China; 4 Key Laboratory of Food Safety Toxicology Research and Evaluation, Beijing 100191, China

**Keywords:** metabolic dysfunction-associated steatotic liver disease, lycopene, mitophagy, PINK1/Parkin, apoptosis

## Abstract

Metabolic dysfunction-associated steatotic liver disease (MASLD) is a prevalent global health concern. Although pharmacotherapies such as Resmetirom and semaglutide have recently gained approval by FDA/EMEA, therapeutic options remain limited, necessitating the exploration of novel natural compounds. Our previous research indicated that lycopene exerts protective effects against MASLD; however, its underlying molecular mechanisms remain incompletely understood. The present study aimed to investigate whether lycopene alleviates MASLD by modulating mitophagy, with a focus on the PINK1/Parkin pathway. C57BL/6J mice were fed with high-fat diet for 12 weeks to induce MASLD and daily gavage of lycopene (10/40 mg/kg). In vitro, AML12 cells were treated with lycopene and Mdivi-1 to assess the role of PINK1/Parkin-mediated mitophagy against lipid accumulation, oxidative stress, and apoptosis. The results found that lycopene supplementation significantly ameliorated HFD-induced weight gain, dyslipidemia, hepatic steatosis, pathological liver injury, and elevated serum liver enzymes. It reduced hepatic reactive oxygen species (ROS) overproduction and suppressed the mitochondrial apoptotic pathway, as evidenced by decreased cytochrome c release and caspase cascade activation. Concurrently, lycopene restored ATP levels and mitochondrial membrane potential, improved ultrastructural integrity, and balanced mitochondrial dynamics by downregulating DRP1 and upregulating MFN2 and OPA1. Crucially, lycopene activated PINK1/Parkin-mediated mitophagy, leading to an increased LC3-II/LC3-I ratio and Beclin1 expression, alongside decreased levels of mitochondrial proteins TOM20 and COX IV. In vitro, the lycopene partially reversed the exacerbating effects of Mdivi-1 on lipid accumulation, ROS generation, apoptosis, and the suppression of the PINK1/Parkin pathway. Collectively, lycopene ameliorates MASLD by activating PINK1/Parkin-mediated mitophagy and improving mitochondrial homeostasis, thereby reducing hepatic lipid accumulation and attenuating hepatocyte apoptosis.

## 1. Introduction

Metabolic dysfunction-associated steatotic liver disease (MASLD), formerly known as nonalcoholic fatty liver disease (NAFLD), has become the most prevalent chronic liver disease worldwide, affecting approximately 30% of the global population [1]. It represents a serious health burden, as it can progress to end-stage liver conditions, including cirrhosis and hepatocellular carcinoma [2]. Moreover, MASLD is strongly associated with a spectrum of metabolic disorders, such as dyslipidemia, type 2 diabetes and hypertension [3]. The disease burden is substantial and continues to escalate; liver-related mortality attributable to MASLD is projected to increase by 178% from 2015 to 2030 [4]. Its steadily increasing prevalence and substantial disease burden pose a significant challenge to public health systems. Although Resmetirom has become the first approved pharmacotherapy for noncirrhotic MASH with advanced fibrosis (F2–F3) [5], and GLP-1 receptor agonists like semaglutide have shown promising efficacy, the latter are primarily indicated for patients with obesity or type 2 diabetes [6]. Consequently, effective treatment options for the broader MASLD spectrum remain scarce, necessitating the identification of novel therapeutic targets and the exploration of promising natural compounds.

Mitochondrial dysfunction is recognized as a central pathogenic mechanism in the progression of MASLD, as delineated by the “multiple hit” hypothesis [7]. This dysfunction leads to excessive production of reactive oxygen species (ROS), which promotes the release of pro-apoptotic factors such as cytochrome c, thereby activating the caspase-mediated apoptotic cascade. The resultant hepatocyte death initiates and amplifies local inflammatory responses, exacerbating liver injury [8]. Furthermore, ROS overproduction directly damages healthy mitochondria, creating a vicious cycle that perpetuates mitochondrial impairment [9]. Efficient clearance of damaged mitochondria is therefore crucial for interrupting this cycle and mitigating disease progression.

Mitophagy, a selective form of autophagy, serves as a key quality-control mechanism by targeting dysfunctional mitochondria for degradation via ubiquitin- and receptor-dependent pathways [10,11]. Mitophagy is orchestrated by several molecular pathways, among which the PINK1/Parkin signaling axis is one of the most well-characterized [12,13]. Accumulating evidence underscores its critical role in MASLD pathogenesis. Studies have shown that impaired PINK1/Parkin-mediated mitophagy is associated with aggravated mitochondrial injury and liver damage in experimental models, whereas pharmacological or genetic activation of this pathway can alleviate MASLD progression [14,15,16,17]. These findings collectively posit the enhancement of PINK1/Parkin-dependent mitophagy as a promising therapeutic strategy for MASLD.

Lycopene, a potent antioxidant carotenoid [18], exhibits diverse pharmacological properties, including anti-inflammatory [19,20], and neuroprotective effects [21]. Our previous study demonstrated its efficacy in preventing high-fat diet (HFD)-induced hepatic steatosis and liver injury [22]; however, the precise molecular mechanisms, particularly concerning mitochondrial quality control, remain to be fully elucidated. Intriguingly, lycopene has been reported to modulate PINK1/Parkin-mediated mitophagy in other pathological contexts. For instance, it alleviated Parkinson’s disease by enhancing this pathway to restore mitochondrial function [23] and protected against splenic toxicity by upregulating a related signaling axis [24]. Despite these indications, direct evidence linking lycopene to PINK1/Parkin activation in MASLD is currently lacking.

Based on the established role of PINK1/Parkin mitophagy in MASLD and the modulatory potential of lycopene, we hypothesized that lycopene protects against MASLD by activating the PINK1/Parkin pathway, thereby enhancing mitophagic clearance of damaged mitochondria and reducing hepatocyte apoptosis. To test this hypothesis, the present study aimed to investigate the effects of lycopene on PINK1/Parkin-mediated mitophagy and its functional consequences in both HFD-fed mice and palmitic acid (PA)-treated AML12 hepatocytes.

## 2. Materials and Methods

### 2.1. Chemicals and Reagents

Lycopene (≥95% purity) was obtained from Yuanye Technology Co., Ltd. (Shanghai, China). The mitophagy inhibitor Mdivi-1 (98.03% purity, molecular weight 353.22 g/mol) was purchased from AbMole (Shanghai, China). PA (sodium palmitate, ≥98.5% purity, Catalog No. P9767, CAS 408-35-5; Sigma-Aldrich, Merck KGaA, Darmstadt, Germany) was used to induce lipo-toxicity in AML12 cells. Serum biochemical parameters, including total cholesterol (TC), triglycerides (TG), low-density lipoprotein-cholesterol (LDL-C), high-density lipoprotein cholesterol (HDL-C), alanine aminotransferase (ALT) and aspartate aminotransferase (AST), were measured using an automatic analyser (Hitachi Instruments Co. Ltd., Tokyo, Japan). Assay kits for free fatty acids (FFA), alkaline phosphatase (ALP), albumin (ALB), total bilirubin (TBIL), superoxide dismutase (SOD), malondialdehyde (MDA), glutathione (GSH) were sourced from the Jiancheng Bioengineering Institute (Nanjing, China). The Tissue Mitochondria Isolation Kit, mitochondrial membrane potential (MMP) assay kit (JC-1), adenosine triphosphate (ATP) assay kit, Oil Red O stain kit, Mitochondrial Superoxide Assay Kit, and MitoTracker Red were obtained from Beyotime Biotechnology (Shanghai, China). All other chemicals were of analytical grade and obtained from commercial suppliers.

### 2.2. Animals and Treatments

#### 2.2.1. Experimental Design

This study was approved by the Laboratory Animal Welfare Ethics Committee of Qingdao University (No. 20240307C576020240617092). All experimental procedures were conducted in strict accordance with the institutional Guidelines for Care and Use of Laboratory Animals.

Fifty 7-week-old male C57BL/6J mice were purchased from Vital River Laboratory Animal Technology (Beijing, China; License No. SCXK 2021-0010). Animals were housed under specific pathogen-free (SPF) conditions at a temperature of 23 ± 1 °C with a 12 h light/dark cycle, and the air exchange rate was maintained at 20 air changes per hour in the Animal Experimentation Center of Qingdao University. Mice were housed five per cage in standard polypropylene cages (M2 model, 320 × 215 × 170 mm), with wooden blocks provided in each cage as environmental enrichment to facilitate natural gnawing and nesting behaviors. Experimental diets were supplied by Jiangsu Xietong Pharmaceutical Bio-engineering Co., Ltd. (Nanjing, China).

Following the previous studies [25,26], a HFD (60% kcal from fat) was administered to C57BL/6J male mice for 12 weeks to induce MASLD model in this study. This 12-week HFD protocol is widely recognized for establishing stable hepatic macrovesicular steatosis [27]. The HFD (XTHF60) provided 60% of energy from fat, 20% from protein and 20% from carbohydrates. The control diet (XTCON50J) provided 10% of energy from fat, 20% from protein and 70% from carbohydrates (detailed formula in Supplementary Table S1).

#### 2.2.2. Rationale for Dose Selection

The dose of lycopene was determined based on previous studies [28,29,30]. Fenofibrate (a clinically used fibrate for lowering triglycerides) was selected as the positive control based on substantial preclinical evidence demonstrating its efficacy against HFD-induced liver injury at 50 mg/kg [31,32,33].

#### 2.2.3. Grouping and Treatment

After a one-week acclimation period, mice were randomly allocated into five groups (*n* = 10 per group): (1) Normal control (NC) group: fed control diet; (2) Model control (MC) group: fed the HFD; (3) Low-dose lycopene (LL) group: fed HFD and administered 10 mg/kg/d lycopene; (4) High-dose lycopene (HL) group: fed HFD and administered 40 mg/kg/d lycopene; (5) Fenofibrate (FN) group: fed HFD and administered 50 mg/kg/d fenofibrate (Sigma-Aldrich, St. Louis, MO, USA). Except for the NC group, all other groups received the HFD for 12 consecutive weeks. Lycopene and fenofibrate were dissolved in soybean oil and administered daily by oral gavage for a duration of 12 consecutive weeks. Mice had libitum access to food and water throughout the 12-week study. Body weight was recorded weekly.

At the end of the 12-week experiment, mice were euthanized following a 12 h fast. After being deeply anesthetized with sodium pentobarbital (50 mg/kg, body weight), mice were subjected to blood collection, followed by cervical dislocation to confirm death. Liver tissue was then excised. All procedures were approved by the Institutional Animal Care and Use Committee (IACUC) of Qingdao University and performed in accordance with the ARRIVE guidelines.

### 2.3. Histopathological Analysis

Hepatic histopathology was evaluated by hematoxylin and eosin (H&E) staining, and lipid deposition was assessed by Oil Red O staining. The detailed procedures were as follows. For H&E staining, liver tissue was fixed in 4% paraformaldehyde, dehydrated, embedded in paraffin, and sectioned at 5 μm thickness. Sections were then stained with hematoxylin and eosin to examine liver histology and morphology. Microscopic observation was performed using a light microscope (Olympus, Tokyo, Japan; 400× magnification). The NAFLD activity score (NAS) was determined based on established criteria, accounting for the degree of steatosis, lobular inflammation, and hepatocyte ballooning [34]. For Oil Red O staining, frozen liver cryosections (5 μm) were counterstained with hematoxylin and subsequently incubated with Oil Red O working solution for 1 h. After incubation, sections were thoroughly rinsed with phosphate-buffered saline (PBS). Stained sections were imaged under a light microscope.

### 2.4. Liver Antioxidant Capacity Testing

Liver tissue samples were homogenized in ice-cold normal saline (1:9, *w*/*v*) and centrifuged at 3000× *g* for 15 min at 4 °C. The supernatant was carefully collected for the measurement of MDA (TBA method), SOD (WST-1 method), and reduced GSH (microplate method) levels using commercial kits (Nanjing Jiancheng Bioengineering Institute, Nanjing, China). The protein concentration of each sample was quantified using a BCA kit (Beyotime, China), and all values were normalized to protein content. All procedures were strictly performed in accordance with the manufacturers’ instructions.

### 2.5. Transmission Electron Microscopy (TEM) Analysis

The ultrastructural features of hepatocyte mitochondria were examined by TEM. Liver tissue samples were cut into 1 mm^3^ cubes and immediately fixed into 2.5% glutaraldehyde at room temperature for 2 h in the dark, followed by storage at 4 °C. After washing with PBS (pH 7.4), samples were post-fixed with 1% osmium tetroxide for 1.5 h at 4 °C. Dehydration was then carried out using a graded acetone series (30–100%). The dehydrated samples were embedded in epoxy resin. Ultrathin sections (70 nm) were cut using an Ultracut E ultramicrotome (Reichert-Jung, Vienna, Austria), collected on copper grids, and stained with 3% uranyl acetate and lead citrate. Finally, the ultrastructure of the liver tissue was observed using a JEM-1200EX transmission electron microscope (JEOL, Tokyo, Japan).

### 2.6. Dihydroethidium (DHE) Staining

Hepatic ROS production was detected by dihydroethidium (DHE) staining. Frozen liver sections were washed three times with PBS and then incubated with 10% goat serum at room temperature for 30 min for blocking. Subsequently, the sections were incubated with 10 μM DHE working solution (D7008, Sigma-Aldrich, St. Louis, MO, USA) for 1 h at 37 °C in the dark. After washing three times with PBS, nuclei were counterstained with 4’,6-diamidino-2-phenylindole (DAPI; G1012, Servicebio, Wuhan, China) for 5 min. Finally, the sections were mounted with an anti-fade mounting medium and visualized under an ECLIPSE C1 fluorescence microscope (NIKON, Tokyo, Japan).

### 2.7. Immunofluorescence Staining

The colocalization of LC3 with the mitochondrial marker TOM20 was assessed by immunofluorescence. Paraffin-embedded liver sections were permeabilized with 0.25% Triton X-100 and blocked with 3% bovine serum albumin (BSA). Sections were then incubated overnight at 4 °C with primary antibodies against TOM20 (1:1000, Servicebio), LC3 (1:500, Servicebio) followed by incubation with appropriate fluorophore-conjugated secondary antibodies for 50 min at room temperature. For the detection of cleaved caspase-3, sections were stained with an anti-cleaved caspase-3 antibody (1:1000, Servicebio). Nuclei were visualized with DAPI. Fluorescent images were captured using a fluorescence microscope.

### 2.8. Cell Culture and Viability Assay

The alpha mouse liver 12 (AML12) cell line was procured from Shanghai Cell Bank (Shanghai, China). Cells were cultured in DMEM/F12 medium (Sparkjade, Jinan, Shandong, China) supplemented with 10% fetal bovine serum (Clark Bioscience, Richmond, VA, USA), 1% ITS liquid medium supplement (Sigma-Aldrich, St. Louis, MO, USA), 1% penicillin-streptomycin (100 U/mL penicillin and 100 μg/mL streptomycin, Logan, UT, HyClone, Logan, UT, USA), and 40 ng/mL dexamethasone. Cells were maintained at 37 °C in a humidified incubator with 5% CO_2_.

To establish the in vitro model and determine intervention doses, cell viability was assessed using the MTT assay. Briefly, AML12 cells seeded in 96-well plates were treated with various concentrations of the respective agents for 0–48 h. After treatment, the medium was replaced with fresh medium containing MTT solution (0.5 mg/mL) which was measured at 490 nm using a microplate reader.

### 2.9. Treatment Concentrations and Durations

Supplementary Figure S1 illustrates the detailed dose- and time-dependent effects of PA, lycopene and Mdivi-1 on AML12 cell viability. PA significantly reduced cell viability in a progressive manner as concentrations (100–500 μM) and incubation times (12–48 h) increased (Supplementary Figure S1A,B, *p* < 0.05). Based on these findings and in accordance with relevant literature [35], 200 μM PA for 24 h was chosen to establish the lipotoxicity model. Lycopene exerted no significant impact on cell viability within the tested ranges (up to 50 μM and 48 h, Supplementary Figure S1C,D, *p* > 0.05), justifying the use of 20 μM as a safe and effective dose. Furthermore, to ensure potent inhibition of mitochondrial fission while mitigating non-specific cytotoxicity, 5 μM Mdivi-1 was selected, as higher concentrations (25–100 μM) resulted in marked, dose-dependent toxicity (Supplementary Figure S1E,F, *p* < 0.05).

### 2.10. Cell Staining

Lipid accumulation, mitochondrial superoxide levels, and mitochondrial morphology in AML12 cells were evaluated using Oil Red O (Beyotime Biotechnology, Shanghai, China), MitoSOX™ Red (Beyotime Biotechnology, Shanghai, China), and MitoTracker Red CMXRos staining (Beyotime Biotechnology, Shanghai, China), respectively. For Oil Red O staining, cells were fixed with 4% paraformaldehyde for 10 min, stained with filtered Oil Red O working solution for 30 min and counterstained with hematoxylin for 2 min. Images were acquired using an inverted fluorescence microscope (Olympus, Japan) at 40× magnification. For detection of mitochondrial superoxide, cells cultured in confocal dishes were incubated with 5 μM MitoSOX™ Red working solution at 37 °C for 20 min, washed twice with PBS, and imaged in PBS using a Leica inverted laser confocal microscope (Leica Microsystems, Wetzlar, Germany). To assess mitochondrial morphology, cells were stained with 200 nM MitoTracker Red CMXRos at 37 °C for 30 min, washed, and then imaged in a fresh pre-warmed culture medium using a Leica inverted laser confocal microscope.

### 2.11. Western Blotting Assay

Subcellular fractionation. Cytosolic and mitochondrial fractions were isolated from liver tissue using a Tissue Mitochondria Isolation Kit (Beyotime Biotechnology, Shanghai, China) according to the manufacturer’s instructions. Briefly, fresh liver samples were washed with ice-cold PBS, minced, and homogenized in mitochondria extraction buffer supplemented with protease and phosphatase inhibitors. The homogenates were centrifuged at 600× *g* for 10 min at 4 °C to remove nuclei and unbroken cells. The resulting supernatant was then centrifuged at 11,000× *g* for 10 min at 4 °C. The supernatant was collected as the cytosolic fraction for subsequent detection of cytochrome c release. The pellet was resuspended in mitochondria lysis buffer and designated as the mitochondria-enriched fraction. The same procedure was applied to AML12 cells using a Cell Mitochondria Isolation Kit (Beyotime Biotechnology, Shanghai, China) to obtain cytosolic and mitochondrial fractions.

For whole-tissue and whole-cell protein extraction, liver tissue and AML12 cells were lysed on ice using RIPA lysis buffer (Beyotime Biotechnology, Shanghai, China) containing protease and phosphatase inhibitors. Protein concentration was determined using a BCA protein assay kit (Beyotime Biotechnology, Shanghai, China). Equal amounts of protein were separated by SDS-PAGE and transferred onto PVDF membranes (Immobilon, Merck KGaA, Darmstadt, Germany). The membranes were blocked with non-fat milk and then incubated overnight at 4 °C with specific primary antibodies (detailed in Supplementary Table S2). After washing, membranes were incubated with appropriate horseradish peroxidase (HRP)-conjugated secondary antibodies for 1 h at room temperature. Protein bands were visualized using an enhanced chemiluminescence (ECL) detection system (Bio-Rad Laboratories, Hercules, CA, USA) and quantified by densitometry using ImageJ software (version 6.0). β-Actin was used as the loading control for normalization.

### 2.12. Statistical Analysis

Data are presented as mean ± standard deviations (SD). Comparisons among multiple groups were performed using one-way analysis of variance (ANOVA), followed by the Least Significant Difference (LSD) post hoc test for pairwise comparisons. In the figures, different lowercase letters indicate significant differences between groups (*p* < 0.05). Quantitative analyses of Oil Red O-positive areas and fluorescence intensity were conducted using ImageJ software (version 6.0). All statistical analyses were conducted with SPSS 23.0 (SPSS Inc, Chicago, IL, USA) and GraphPad Prism 8.0 (GraphPad software, San Diego, CA, USA). A two-tailed *p* value < 0.05 was considered statistically significant.

## 3. Results

### 3.1. Lycopene Alleviates Obesity and Lipid Metabolism Disorders in HFD-Fed Mice

**Figure 1:**
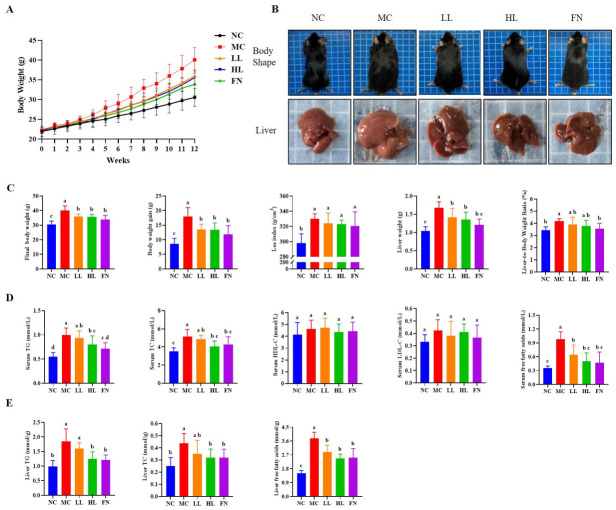
Lycopene ameliorates HFD-induced adiposity and lipid metabolic disorders. (**A**) Dynamic changes in body weight of mice. (**B**) Representative photographs of mice body shape and liver. (**C**) Final body weight, body weight gain, Lee index, liver weight, and liver-to-body weight ratio of mice at termination of study. (**D**) Serum levels of total triglyceride (TG), cholesterol (TC), high-density lipoprotein cholesterol (HDL-C), low-density lipoprotein cholesterol (LDL-C), and free fatty acid (FFA) in mice. (**E**) Liver levels of TG, TC and FFA. NC: Normal control group; MC: Model control group; LL: Low-dose lycopene group, HL: High-dose lycopene group, FN: Fenofibrate group. Different lowercase letters indicate significant differences between groups (*p* < 0.05).

As illustrated in Figure 1A, mice in the MC group gained body weight more rapidly than those in the NC group, with a statistically significant divergence emerging from the fifth week onward. Lycopene administration attenuated the HFD-induced weight gain, with the attenuation appearing more evident at the higher dose. After 12 weeks, MC group mice exhibited a significantly obese phenotype, whereas lycopene-treated mice exhibited a leaner body contour (Figure 1B). Consistent with these observations, the final body weight, body weight gain, liver weight were all significantly lower in both LL and HL groups compared to the MC group (*p* < 0.05) (Figure 1C). While the Lee’s index and the liver-to-body weight ratio showed no statistically significant differences compared to the MC group (*p* > 0.05). The effects of high and low doses of lycopene on these indices were comparable (*p* > 0.05), and there was no statistical difference between them and the fenofibrate group (*p* > 0.05).

Serum levels of TG, TC and FFA, as well as hepatic TG, TC and FFA content, were significantly elevated in the MC group compared to the NC group (*p* < 0.05), while serum HDL-C and LDL-C levels remained unchanged (*p* > 0.05). Supplementation with lycopene for 12 weeks significantly reduced these HFD-induced elevations in both serum and hepatic lipid profiles (*p* < 0.05) (Figure 1D,E), with the HL group demonstrating a more pronounced effect than the LL group in lowering hepatic TG content (*p* < 0.05).

### 3.2. Lycopene Attenuated Pathological and Liver Function Abnormalities in HFD-Fed Mice

Macroscopic examination revealed that livers from the NC group were small, ruddy in color, and hard sharp edges. In contrast, livers from the MC group were markedly enlarged, exhibited an earthy yellow discoloration, dull edges, and tense capsules with visible lipid droplets (Figure 1B). Lycopene treatment reduced liver volume and improved both color and surface luster compared to the MC group.

**Figure 2:**
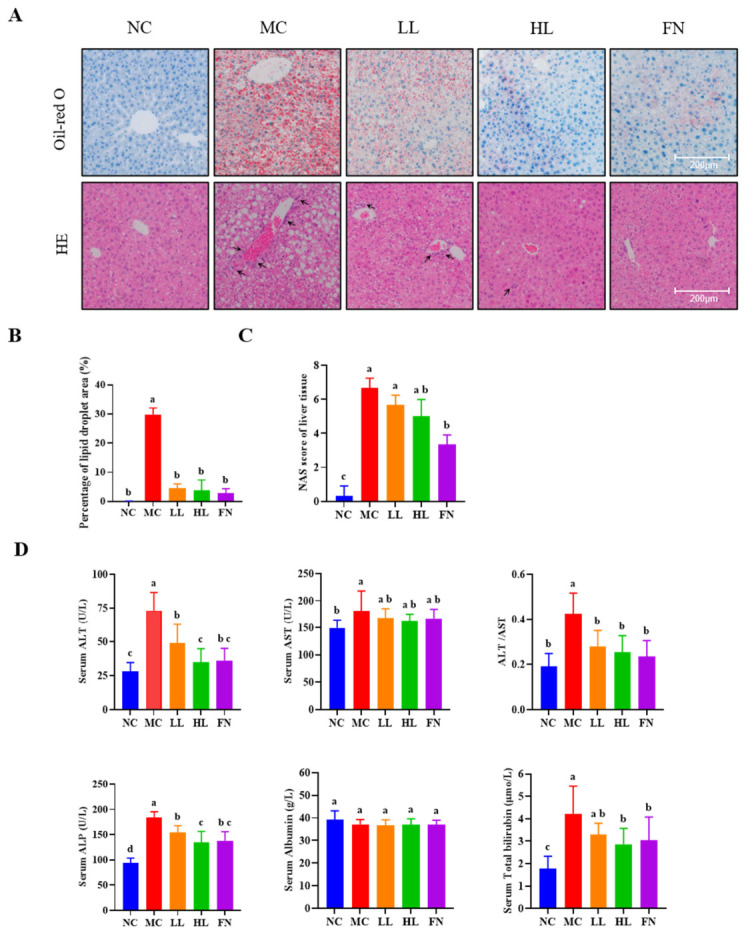
Lycopene ameliorates liver injury and lipid accumulation of mice. (**A**) Representative images of Oil Red O and H&E staining of the liver. (**B**) Percentage of lipid droplets area of Oil Red O (%) (*n* = 3). (**C**) NAS of liver tissue (*n* = 3). (**D**) The serum levels of ALT, AST, ALT/AST, ALP, Albumin and Total bilirubin. NC: Normal control group; MC: Model control group; LL: Low-dose lycopene group, HL: High-dose lycopene group, FN: Fenofibrate group. Black arrows indicate inflammatory cell infiltration. Different lowercase letters indicate significant differences between groups (*p* < 0.05).

Histological analysis corroborated these findings. Oil Red O staining demonstrated extensive hepatic lipid deposition in the MC group, characterized by numerous cytoplasmic lipid droplets of varying sizes. Lycopene intervention, particularly at the high dose, substantially ameliorated this steatosis (Figure 2A). Quantitative analysis confirmed that the Oil Red O-positive area was significantly smaller in the LL and HL groups than in the MC group (*p* < 0.05) (Figure 2B), and the efficacy of the HL group was closer to the FN group. H&E staining further revealed that HFD feeding disrupted hepatic lobule architecture, induced significant steatosis, and promoted lobular inflammation. Lycopene supplementation, particularly in the HL group, restored hepatic cord arrangement and lobule structure, attenuated steatosis, and reduced inflammatory cell foci. Accordingly, the NAS was significantly lower in higher dose lycopene-treated group than in the MC group (*p* < 0.05) (Figure 2C).

Serum biochemistry revealed pronounced liver injury in the MC group, with significantly elevated levels of ALT, AST, ALT/AST ratio, ALP, and TBIL compared to the NC group (*p* < 0.05) (Figure 2D). Lycopene treatment significantly attenuated the HFD-induced increases in ALT, ALT/AST, ALP, and TBIL (*p* < 0.05), with the efficacy of high-dose lycopene being comparable to that of the positive control fenofibrate (50 mg/kg).

### 3.3. Lycopene Reduces Hepatic ROS Accumulation and Attenuates Oxidative Stress in HFD-Fed Mice

**Figure 3:**
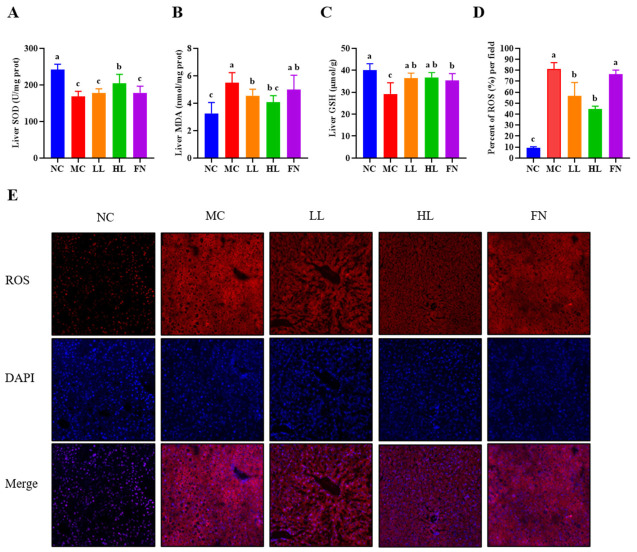
Lycopene inhibits ROS to improve the antioxidant power of mice liver. (**A**–**D**) Biochemical measurements of SOD (**A**), MDA (**B**), GSH (**C**), mean density of ROS (**D**) in liver tissue (*n* = 3). (**E**) Immunofluorescence of ROS. NC: Normal control group; MC: Model control group; LL: Low-dose lycopene group, HL: High-dose lycopene group, FN: Fenofibrate group. Different lowercase letters indicate significant differences between groups (*p* < 0.05).

To elucidate the impact of lycopene on hepatic oxidative stress, we measured the activities of antioxidant enzymes and the levels of oxidative damage markers. Compared with the NC group, the MC group exhibited significantly decreased activities of SOD and GSH, along with a significant increase in MDA content (*p* < 0.05) (Figure 3A–C). Lycopene treatment significantly reversed these changes, restoring SOD and GSH activities and reducing MDA levels (*p* < 0.05). The effects of high and low doses of lycopene on MDA and GSH were comparable; moreover, lycopene exhibited enhanced efficacy over fenofibrate in counteracting HFD-induced oxidative stress in the SOD level.

We next assessed intrahepatic ROS levels using DHE staining (Figure 3D,E). Hepatic sections from the MC group exhibited a significant increase in red fluorescence intensity compared to the NC group (*p* < 0.05), indicating substantial ROS accumulation. Both LL and HL treatments significantly attenuated this fluorescence signal compared to the MC group, with the HL group demonstrating a more potent ROS-scavenging effect trend.

### 3.4. Lycopene Inhibits HFD-Induced Hepatocyte Apoptosis

**Figure 4:**
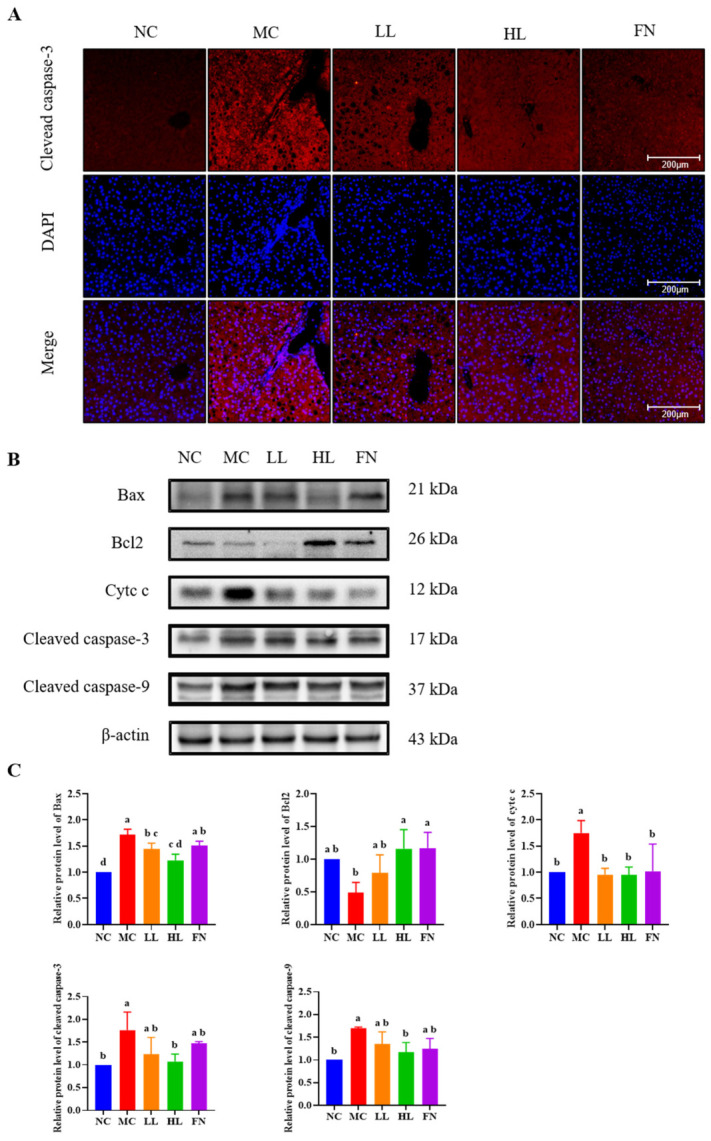
Effects of lycopene on apoptosis-related regulatory elements in the livers of mice. (**A**) Immunofluorescence of cleaved caspase-3. The cleaved caspase-3 was stained with red, and nuclei were labeled with blue. (**B**,**C**) Western blotting detected the expression of Bax, Bcl2, Cytc c, cleaved caspase-3, and cleaved caspase-9. NC: Normal control group; MC: Model control group; LL: Low-dose lycopene group, HL: High-dose lycopene group, FN: Fenofibrate group. Different lowercase letters indicate significant differences between groups (*p* < 0.05).

To assess apoptosis, we first examined the activation of cleaved caspase-3, a key executioner protease, by immunofluorescence (Figure 4A). A pronounced increase in cleaved caspase-3 was observed in the livers of MC group compared to the NC group. Lycopene treatment effectively attenuated this caspase-3 activation.

We further analyzed the expression of apoptosis-related proteins by Western blotting (Figure 4B,C). Compared with the NC group, the MC group showed significant upregulation of the pro-apoptotic proteins Bax, cytosolic cytochrome c, cleaved caspase-3, and cleaved caspase-9, alongside downregulation of the anti-apoptotic protein Bcl2 (*p* < 0.05). Lycopene intervention significantly reversed these changes, downregulating Bax, cytochrome c, cleaved caspase-3, and cleaved caspase-9, while upregulating Bcl2 expression (*p* < 0.05).

### 3.5. Lycopene Ameliorates Mitochondrial Dysfunction in HFD-Fed Mice

**Figure 5:**
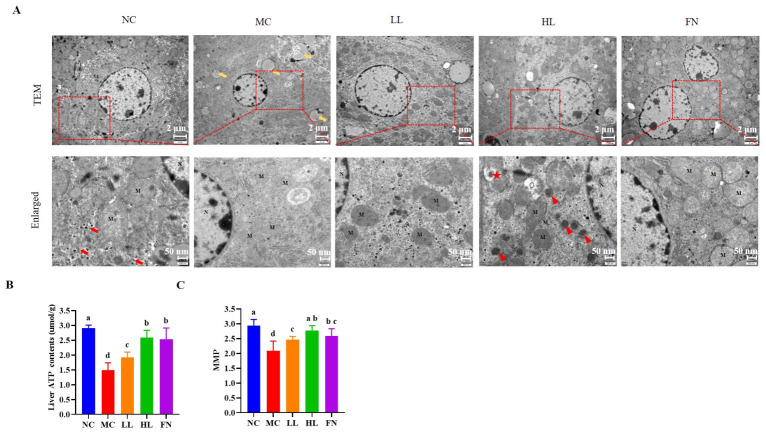
Lycopene restored mitochondrial function in the mice liver. (**A**) Transmission electron microscopy of mice liver. (**B**) ATP contents in liver tissue. (**C**) Mitochondrial membrane potential level. M: mitochondria; N: nucleus; yellow arrows: lipid droplets; red arrows: autolysosomes; red triangles: nascent mitophagosomes; red stars: mitophagosomes. NC: Normal control group; MC: Model control group; LL: Low-dose lycopene group, HL: High-dose lycopene group, FN: Fenofibrate group. Different lowercase letters indicate significant differences between groups (*p* < 0.05).

TEM analysis of hepatocyte ultrastructure revealed severe mitochondrial damage in the MC group, characterized by mitochondrial swelling, vacuolization, and an increased number of fragmented organelles (Figure 5A). The number of autophagosomes was also reduced compared to the NC group. Lycopene treatment restored regular mitochondrial architecture, increased the number of intact mitochondria with clearly visible cristae, and promoted the formation of autolysosomes.

Consistent with the morphological improvements, functional assessments showed that MMP and ATP content were significantly lower in the MC group than those in the NC group (Figure 5B,C) (*p* < 0.05). Lycopene treatment significantly restored MMP and ATP levels (*p* < 0.05), with the high dose restoring them to near-normal levels.

### 3.6. Lycopene Activates the PINK1/Parkin Mitophagy Pathway and Restores Mitochondrial Dynamics in Mouse Livers

**Figure 6:**
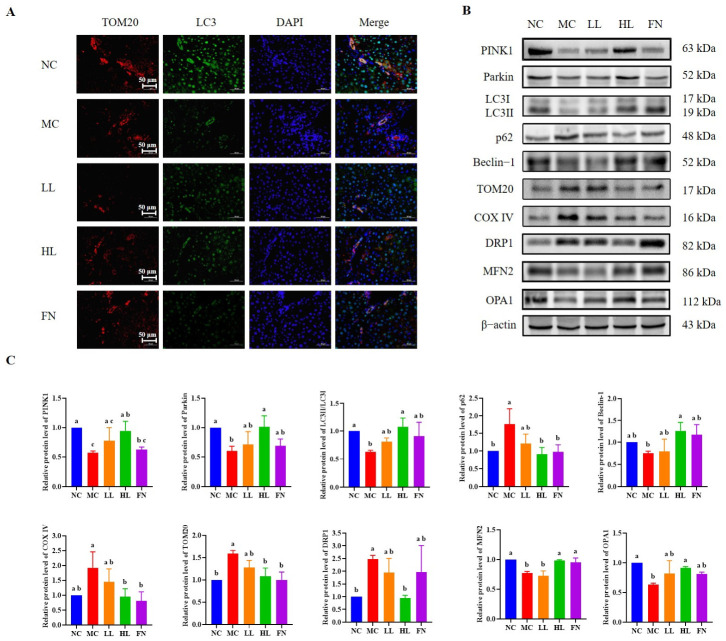
Lycopene activates the mitophagy pathway PINK1/Parkin and alters mitochondrial dynamics. (**A**) Representative immunofluorescence confocal microscopy images of LC3 (green) and TOM20 (red) staining of hepatocytes. (**B**,**C**) Western blotting detected the expression of PINK1, Parkin, LC3II/LC3I, p62, Beclin-1, TOM20, COX IV, DRP1, MFN2 and OPA1. NC: Normal control group; MC: Model control group; LL: Low-dose lycopene group, HL: High-dose lycopene group, FN: Fenofibrate group. Different lowercase letters indicate significant differences between groups (*p* < 0.05).

Western blot analysis revealed significant downregulation of PINK1 and Parkin expression in the MC group compared to the NC group (*p* < 0.05), along with a decreased LC3-II/LC3-I ratio and Beclin-1 level, concomitant with increased accumulation of p62 and elevated levels of the mitochondrial proteins TOM20 and COX IV (*p* < 0.05). High-dose lycopene treatment effectively reversed these alterations, upregulating PINK1, Parkin, the LC3-II/LC3-I ratio, and Beclin-1, while downregulating p62, TOM20, and COX IV (*p* < 0.05) (Figure 6B,C). To corroborate these findings and specifically assess mitophagy occurrence, we performed immunofluorescence co-staining of LC3 and TOM20. As shown in Figure 6A, treatment with lycopene significantly increased the colocalization of LC3 (autophagosome marker) with TOM20 (mitochondrial marker), indicating enhanced mitophagic activity.

We further investigated the effects of lycopene on mitochondrial dynamics, a process closely linked to mitophagy (Figure 6B,C). The expression of the fission protein DRP1 was significantly increased in the MC group compared to the NC group (*p* < 0.05), whereas high-dose lycopene treatment markedly suppressed this elevation (*p* < 0.05). Conversely, the levels of fusion proteins MFN2 and OPA1 were decreased in the MC group, and high-dose lycopene intervention significantly restored their expression (*p* < 0.05). This rebalancing of mitochondrial dynamics likely contributes to the attenuation of excessive mitochondrial fragmentation.

### 3.7. Lycopene’s Protection Against MASLD Is Dependent on PINK1/Parkin-Mediated Mitophagy In Vitro

**Figure 7:**
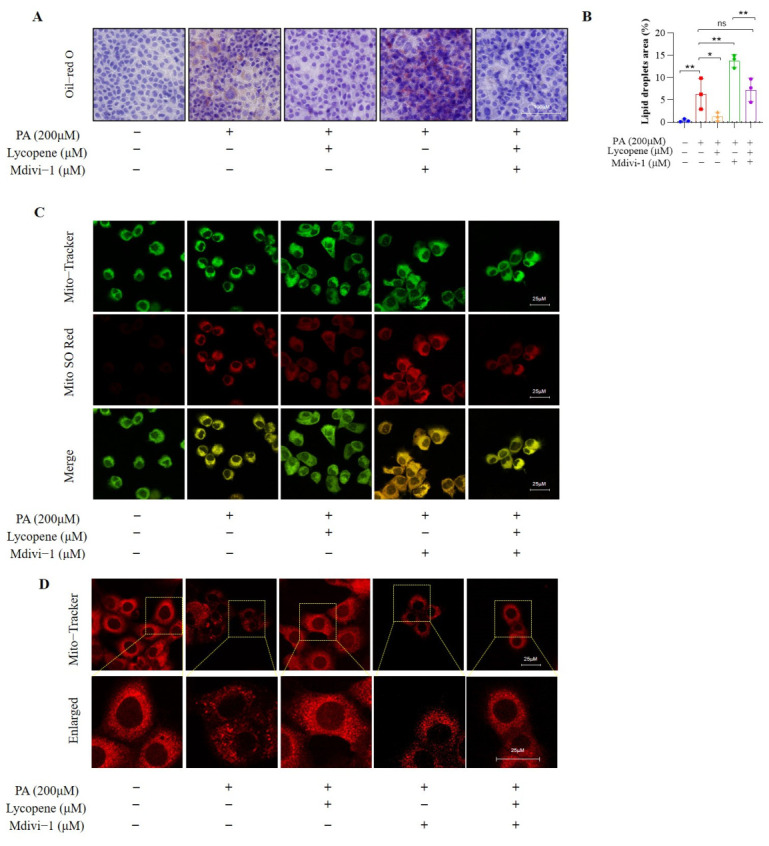
Lycopene alleviate palmitic acid-induced hepatocyte lipotoxicity and mitochondrial dysfunction by improving mitophagy. (**A**) Oil Red O staining showing lipid accumulation in different groups, 50×. (**B**) Lipid droplets area of Oil Red O staining. (**C**) Mitochondria-ROS co-staining revealing oxidative stress levels. The red fluorescence from MitoSOX-Red indicates the production of mitochondrial ROS, with brighter red fluorescence indicating higher oxidative stress. (**D**). Mitochondrial superoxide production was assessed by MitoSOX™ Red staining (red fluorescence). Mitochondria were counterstained with Mito-Tracker™ (green fluorescence). Colocalization of MitoSOX™ Red and Mito-Tracker™ is shown in the merged images. Representative immunofluorescence images of cellular mitochondrial morphology; * *p* < 0.05, ** *p* < 0.001.

To establish a causal link between the observed benefits and mitophagy, we employed the specific inhibitor Mdivi-1 in AML12 cells. As anticipated, co-treatment with Mdivi-1 exacerbated PA-induced lipid accumulation, whereas lycopene partially reversed this aggravation (Figure 7A,B). Similarly, lycopene co-treatment mitigated PA-induced ROS generation and counteracted the further increase in ROS caused by Mdivi-1 (Figure 7C). Moreover, lycopene alleviated PA-induced mitochondrial fragmentation, as well as the exacerbated fragmentation caused by Mdivi-1 co-treatment (Figure 7D).

**Figure 8:**
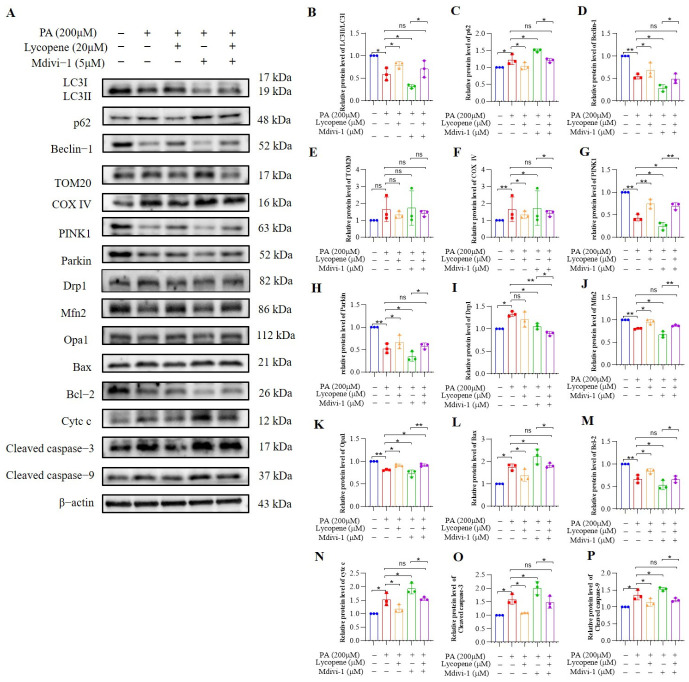
Lycopene restore autophagy flux and mitigate PA-induced mitochondrial dysfunction and apoptosis in hepatocytes in vitro. (**A**) Representative Western blot images of proteins related to mitophagy, mitochondrial dynamics, and apoptosis. (**B**) Quantitative analysis of LC3II/LC3I. (**C**) Quantitative analysis of p62 protein expression. (**D**) Quantitative analysis of Beclin-1 protein expression. (**E**) Quantitative analysis of TOM20 protein expression. (**F**) Quantitative analysis of COX IV protein expression. (**G**) Quantitative analysis of PINK1 protein expression. (**H**) Quantitative analysis of Parkin protein expression. (**I**) Quantitative analysis of Drp1 protein expression. (**J**) Quantitative analysis of Mfn2 protein expression. (**K**) Quantitative analysis of Opa1 protein expression. (**L**) Quantitative analysis of Bax protein expression. (**M**) Quantitative analysis of Bcl-2 protein expression. (**N**) Quantitative analysis of cytc c protein expression. (**O**) Quantitative analysis of Cleaved caspase-3 protein expression. (**P**) Quantitative analysis of Cleaved caspase-9 protein expression. Bars in distinct colors represent different experimental groups. * *p* < 0.05, ** *p* < 0.001.

Western blot analysis of the apoptotic pathway showed that PA treatment upregulated Bax, cytosolic cytochrome c, cleaved caspase-9, and cleaved caspase-3, while downregulating Bcl2 (Figure 8A,B). Mdivi-1 exacerbated these PA-induced pro-apoptotic changes, and notably, lycopene significantly reversed the effects of Mdivi-1 (*p* < 0.05), suggesting that its anti-apoptotic action is partially dependent on mitophagy.

Finally, we examined key proteins in the PINK1/Parkin-mediated mitophagy pathway (Figure 8A,B). PA treatment suppressed mitophagy, as shown by a decreased LC3-II/LC3-I ratio and Beclin-1 level, alongside increased p62, TOM20, and COX IV (*p* < 0.05). Mdivi-1 further inhibited this pathway. Crucially, lycopene co-treatment partially counteracted the Mdivi-1-induced suppression of PINK1, Parkin, LC3-II/LC3-I ratio, and Beclin-1, and downregulated the expression of p62, TOM20, and COX IV (*p* < 0.05).

## 4. Discussion

This study provided novel mechanistic insights into the protective effects of lycopene against MASLD. We demonstrated for the first time that lycopene ameliorates hepatic steatosis, oxidative stress, and apoptosis by activating PINK1/Parkin-mediated mitophagy and restoring mitochondrial homeostasis. In vivo, lycopene balanced mitochondrial dynamics, mitophagic flux, and suppressed the intrinsic apoptotic pathway. In vitro, the protective effects of lycopene were partially abrogated by the mitophagy inhibitor Mdivi-1, underscoring the functional dependency of its benefits on this quality-control pathway.

Several nutraceuticals (e.g., quercetin [13], hesperetin [14], and cyanidin-3-O-glucoside [15]) ameliorate MASLD by modulating mitophagy. As a lipophilic compound that preferentially distributes to the liver, lycopene is able to regulate mitochondrial quality control. It rebalances mitochondrial dynamics (DRP1/MFN2/OPA1), thereby achieving efficient PINK1/Parkin-mediated mitophagic clearance. Importantly, unlike previous associative studies, we established a causal relationship using the pharmacological inhibitor Mdivi-1.

The bioavailability of lycopene is a key determinant of its pharmacological efficacy. Lycopene bioavailability is typically estimated to be between 10% and 30%, which is greatly affected by food substrates and dietary components [36]. In our study, lycopene was dissolved in soybean oil and administered by oral gavage, an optimal lipid vehicle that enhances micellarization and lymphatic transport, thereby ensuring sufficient hepatic exposure [37]. Although serum or liver lycopene levels were not quantified in this study due to limited sample availability, studies in rodents have shown that lycopene preferentially accumulates in the liver, reaching concentrations over 10-fold higher than those observed in other tissues [38]. Notably, lycopene exhibits a remarkable enrichment in hepatic mitochondria, where its concentration can be 3- to 5-fold higher than in total liver homogenates [39]. This subcellular distribution pattern provides a structural basis for lycopene to directly modulate mitochondrial quality control pathways.

The pivotal finding of this study is the identification of PINK1/Parkin-mediated mitophagy as a central mechanism through which lycopene exerts its hepatoprotective effects. In MASLD, the efficiency of this quality-control pathway is compromised, leading to the accumulation of dysfunctional mitochondria [40]. Emerging evidence highlights the diverse roles of carotenoids in modulating mitochondrial homeostasis. For instance, astaxanthin alleviates NAFLD-associated mitochondrial dysfunction primarily via the FGF21/PGC-1α axis [41], whereas fucoxanthin promotes Parkin-mediated mitophagy in extrahepatic tissues, such as the retina and myocardium [42,43]. Distinctively, lycopene exhibits superior lipophilicity and preferential hepatic accumulation. Our study fills a crucial gap by establishing that lycopene reactivates the PINK1/Parkin-mediated mitophagy to combat MASLD. We demonstrated that lycopene treatment robustly reactivated this suppressed pathway, as evidenced by the upregulation of PINK1 and Parkin, increased conversion of LC3-I to LC3-II and Beclin-1 expression, alongside the clearance of p62 and mitochondrial substrates (TOM20, COX IV). This restorative effect aligns with genetic evidence. Specifically, PINK1 overexpression in HFD-fed mice restored ATP levels and mitochondrial membrane potential, reduced lipid droplet area by approximately 38%, and significantly lowered serum ALT/AST levels [44]. Conversely, Parkin knockout exacerbated HFD-induced hepatic steatosis, increasing Oil Red O-positive area by 45% and elevating ALT/AST by 1.7-fold [45]. Intriguingly, lycopene’s modulation of this axis is context-dependent, enhancing mitophagy in Parkinson’s models [23] but suppressing its overactivation in certain toxicological contexts [46,47]. This suggests that lycopene does not merely act as a universal mitophagy inducer, but rather as a homeostatic regulator that fine-tunes mitochondrial turnover to match pathophysiological demand.

The hepatoprotection conferred by lycopene is intrinsically linked to its suppression of mitochondrial-initiated apoptosis. Excessive lipid accumulation overloads mitochondrial β-oxidation [48], leading to electron transport chain dysfunction, ROS overproduction, and oxidative stress [49]. In our study, HFD-fed mice exhibited elevated hepatic ROS and MDA alongside depleted antioxidant defenses (SOD, GSH). This finding corroborates prior evidence from human and animal studies [50,51]. This milieu triggers mitochondrial outer membrane permeabilization, facilitating cytochrome c release and subsequent activation of the caspase cascade [52]. Lycopene effectively attenuated this cascade by reducing oxidative stress and modulating the Bcl2/Bax balance, thereby curtailing hepatocyte apoptosis—a pivotal event in MASLD progression [53,54].

Mitochondrial dynamics are intimately coupled with mitophagy. Clinical biopsies from MASLD patients consistently exhibit an imbalance favoring DRP1-mediated fission, which correlates with disease severity [55]. Our model recapitulated this feature, as HFD-fed mice displayed a fission-dominant state (increased DRP1, decreased MFN2/OPA1), aligning with studies linking steatosis to excessive fragmentation [56,57]. Lycopene intervention rebalanced this dynamic by downregulating DRP1 and upregulating MFN2 and OPA1. Importantly, controlled fission is a prerequisite for segregating and targeting damaged mitochondria for PINK1/Parkin-mediated degradation [58,59]. Therefore, lycopene’s regulation of dynamics likely establishes the necessary structural context for efficient mitophagy, representing a coordinated upstream event in mitochondrial quality control.

The causal link between lycopene’s benefits and mitophagy was decisively established through targeted inhibition. Using Mdivi-1, a known suppressor of DRP1-dependent fission [60,61,62,63], we demonstrated that impairing this pathway abolished a significant portion of lycopene’s efficacy. Lycopene’s ability to attenuate PA-induced lipid accumulation, ROS overproduction, and apoptosis was markedly compromised in the presence of Mdivi-1. This observation aligns with the findings of Su et al., who reported that Mdivi-1 neutralized the neuroprotective benefits of resveratrol in HFD-induced cognitive dysfunction [64]. Notably, Mdivi-1’s primary action on mitochondrial fission (Drp1) means that the observed effects are due to blocking the mitochondrial fission prerequisite for mitophagy, rather than direct pharmacological antagonism of PINK1/Parkin binding.

Collectively, our findings support a novel, integrated mechanistic model: Lycopene ameliorates MASLD by (1) first rebalancing mitochondrial dynamics, curbing excessive fission and promoting fusion; (2) this restructured mitochondrial network facilitates the efficient recognition and tagging of damaged organelles via the PINK1/Parkin pathway; (3) the consequent activation of mitophagy enables the selective clearance of these dysfunctional mitochondria, which (4) mitigates mitochondrial ROS overproduction, prevents cytochrome c release, and thereby suppresses the intrinsic apoptotic cascade, ultimately alleviating hepatocyte death and liver injury.

Our study has certain limitations. Mitophagy is a dynamic process, and our snapshot measurements do not fully capture its flux in real-time. Furthermore, while we focused on the canonical PINK1/Parkin pathway, other mitophagy receptors (e.g., FUNDC1, BNIP3) may also contribute to lycopene’s effects [65]. The precise upstream signaling cascades and the direct molecular target through which lycopene initiates mitochondrial quality control and the PINK1/Parkin axis remain to be fully elucidated, warranting further investigation. Additionally, AML12 cells may not fully recapitulate the responses of primary hepatocytes, warranting future validation of our in vitro findings. Finally, further clinical trials in patients with MASLD are needed to validate these findings.

## 5. Conclusions

In conclusion, lycopene ameliorates MASLD by activating PINK1/Parkin-mediated mitophagy to restore mitochondrial homeostasis. This is achieved through rebalancing mitochondrial dynamics (downregulated DRP1 and upregulated MFN2/OPA1), enhancing mitophagic flux (increased LC3 II/LC3 I ratio and Beclin1, decreased TOM20 and COX IV), and suppressing intrinsic apoptosis (reduced cytochrome c release and caspase-9/3 activation), which were partially counteracted by Mdivi-1. Our findings reveal a novel mechanism for lycopene’s hepatoprotective action and support its potential as a nutraceutical strategy for MASLD.

## Data Availability

The original contributions presented in this study are included in the article/Supplementary Materials. Further inquiries can be directed to the corresponding authors.

## Supplementary Materials

The following supporting information can be downloaded at: https://www.mdpi.com/article/10.3390/antiox15050648/s1. Supplementary Figure S1. Effects of PA, lycopene, and Mdivi-1 on AML12 cell viability. Dose- and time-dependent responses were assessed by CCK-8 assay. Data are shown as mean ± SD (*n* = 6). Ns, not significant; * *p* < 0.05; **** *p* < 0.0001. Supplementary Table S1. Ingredient composition and energy distribution of the experimental diets. Supplementary Table S2. The detailed information of primary antibodies used for WB.

## Abbreviations

The following abbreviations are used in this manuscript:

ALB	Albumin
ALP	Alkaline phosphatase
ALT	Alanine aminotransferase
ANOVA	One-way analysis of variance
AST	Aspartate aminotransferase
ATP	Adenosine triphosphate
BSA	Bovine serum albumin
DHE	Dihydroethidium
FFA	Free fatty acids
GSH	Glutathion
HDL-C	High-density lipoprotein cholesterol
H&E	Hematoxylin and eosin
HFD	High-fat diet
LDL-C	Low-density lipoprotein-cholesterol
LSD	Least Significant Difference
MDA	Malondialdehyde
MMP	Mitochondrial membrane potential
MASLD	Metabolic dysfunction-associated steatotic liver disease
NAFLD	Nonalcoholic fatty liver disease
NAS	NAFLD activity score
PA	Palmitic acid
PBS	Phosphate buffered saline
ROS	Reactive oxygen species
SD	Standard deviations
SOD	Superoxide dismutase
TBIL	Total bilirubin
TC	Total cholesterol
TEM	Transmission electron microscopy
TG	Triglycerides
