# Supplementary material for: Lycopene Ameliorates Metabolic Dysfunction-Associated Steatotic Liver Disease via PINK1/Parkin-Mediated Mitophagy Activation and Apoptosis Attenuation
Source: Antioxidants (Basel). 2026 May 21;15(5):648. doi: 10.3390/antiox15050648 (PMC13203502; doi:10.3390/antiox15050648)
Supplement: Supplementary file 1 [file antioxidants-15-00648-s001.zip › antioxidants-4262642-supplementary.pdf]

Supplementary Materials

Supplementary Figure S1. Effects of PA, lycopene, and Mdivi-1 on AML12 cell viability. Dose- and time-dependent responses were assessed by CCK-8 assay. Data are shown as mean ± SD (n=6). Ns, not significant; *$p < 0.05$; ****$p < 0.0001$.

Supplementary Table S1. Ingredient composition and energy distribution of the experimental diets.

Supplementary Table S2. The detailed information of primary antibodies used for WB.

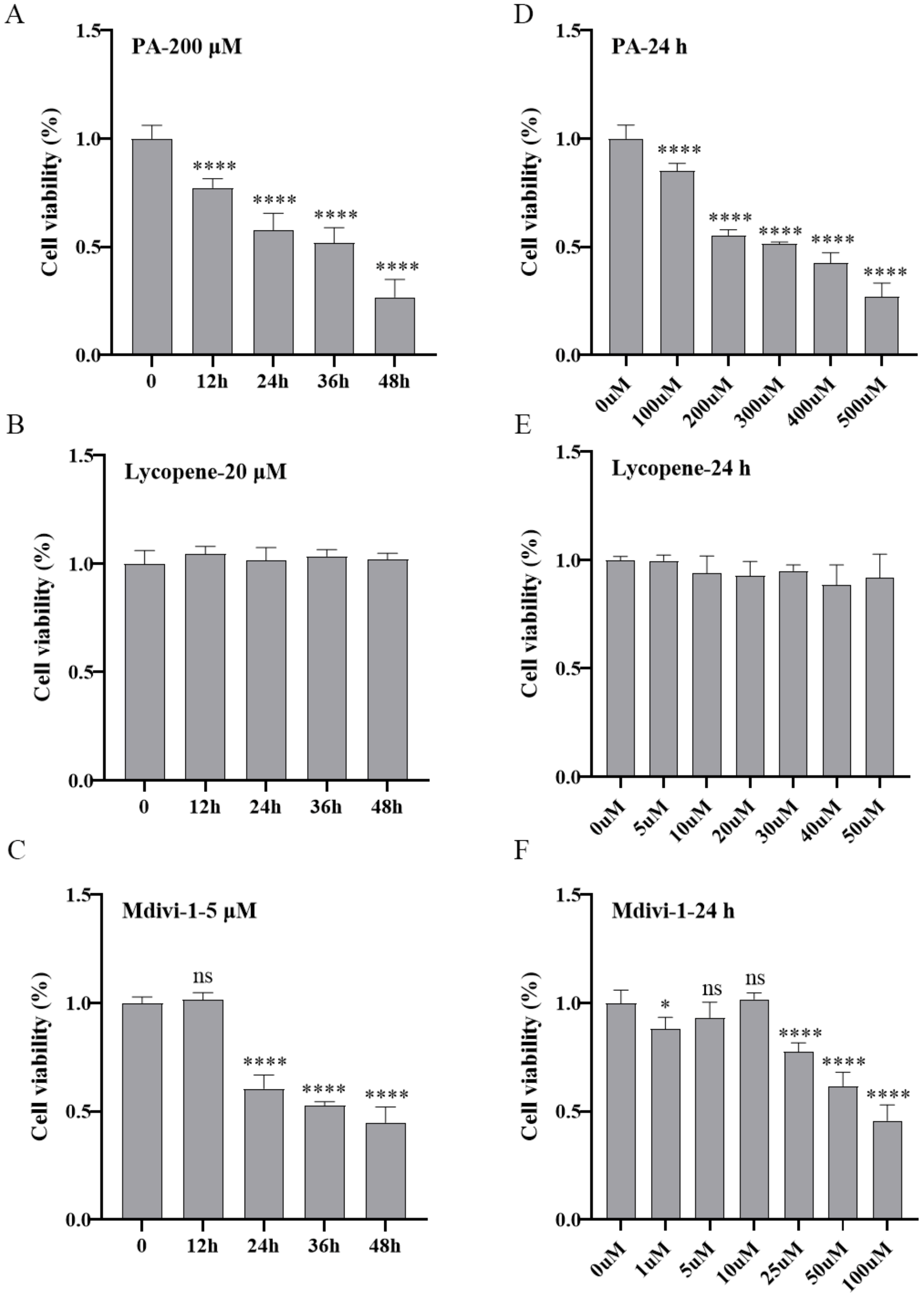

Supplementary Figure S1. Effects of PA, lycopene, and Mdivi-1 on AML12 cell viability. Dose- and time-dependent responses were assessed by CCK-8 assay. Data are shown as mean ± SD (n=6). Ns, not significant; **p* < 0.05; *****p* < 0.0001

Supplementary Table S1. Ingredient composition and energy distribution of the experimental diets.

	**XTHF60 (High-fat diet)**		**XTCON50J (Control diet)**	
**Product**				
Ingredient	gm	kcal	gm	kcal
Casein	200.0	800.0	200.0	800.0
L-Cystine	3.0	12.0	3.00	12.0
Corn Starch	0.0	0.0	506.2	2024.8
Maltodextrin	125.0	500.0	125.0	500.0
Sucrose	72.8	291.2	72.8	291.2
Cellulose	50.0	0.0	50.0	0.0
Soybean Oil	25.0	225.0	25.0	225.0
Lard	245.0	2205.0	20.0	180.0
Mineral Mix S10026B	50.0	0.0	50.0	0.0
Vitamin Mix V10001C	1.0	4.0	1.0	4.0
Choline Bitartrate	2.0	0.0	2.0	0.0
FD&C Blue Dye #1	0.05	0.0	0.01	0.0
FD&C Yellow Dye #5	0.0	0.0	0.04	0.0
Total	773.85	4037.2	1055.05	4037.0
**Energy supply composition**	Quality (gm)%	Supply of energy (kcal)%	Quality (gm)%	Supply of energy (kcal)%
Protein	26.0	20.0	19.2	20.0
Fat	35.0	60.0	4.3	10.0
Carbohydrate	26.0	20.0	67.3	70.0

Supplementary Table S2. The detailed information of primary antibodies used for WB.

Antibody	Producers	Cataloguenumber	Source	Dilution
LC3	Abcam	EPR18709	Rabbit	1:2000
p62	Proteintech	29503-1-AP	Rabbit	1:2000
Beclin-1	Abcam	EPR19662	Rabbit	1:2000
TOM20	Cell Signaling Technology	42406	Rabbit	1:2000
COX IV	Cell Signaling Technology	4850	Rabbit	1:1000
PINK1	Proteintech	81991-4-RR	Rabbit	1:2000
Parkin	Proteintech	14060-1-AP	Rabbit	1:2000
DRP1	Cell Signaling Technology	8570	Rabbit	1:2000
MFN2	Proteintech	12186-1-AP	Rabbit	1:5000
OPA1	Proteintech	27733-1-AP	Rabbit	1:1000
Bax	Proteintech	50599-2-Ig	Rabbit	1:2000
Bcl2	Proteintech	82469-6-RR	Rabbit	1:2000
Cleaved caspase-3	Abcam	EPR21032	Rabbit	1:5000
Cleaved caspase-9	Cell Signaling Technology	9509	Rabbit	1:1000
Cytc c	Proteintech	83276-1-RR	Rabbit	1:5000
β-Actin	Servicebio	GB15003	Rabbit	1:1000
